# Antiretroviral Therapy and Reasons for Not Taking It among Men Having Sex with Men (MSM)—Results from the European MSM Internet Survey (EMIS)

**DOI:** 10.1371/journal.pone.0121047

**Published:** 2015-03-20

**Authors:** Ulrich Marcus, Ford Hickson, Peter Weatherburn, Martina Furegato, Michele Breveglieri, Rigmor C. Berg, Axel J. Schmidt, for the EMIS network

**Affiliations:** 1 Department of Infectious Disease Epidemiology, Robert Koch Institute, Berlin, Germany; 2 Sigma Research, London School of Hygiene & Tropical Medicine, London, United Kingdom; 3 Regione del Veneto, Department of Health, Verona, Italy; 4 Norwegian Knowledge Centre for the Health Services, Oslo, Norway; David Geffen School of Medicine at UCLA, UNITED STATES

## Abstract

**Background:**

The preventive effects of antiretroviral treatment (ART) on onward transmission of HIV are a major reason for broadening eligibility for ART. In the WHO European Region, surveillance reveals substantial differences in access to ART across regions and sub-populations. We analysed self-reported data on ART and reasons for not taking ART from EMIS, a large Pan-European Internet survey among men-who-have-sex-with-men (MSM).

**Methods:**

Respondents from 38 European countries reported their last HIV test result and, if diagnosed with HIV, their treatment status, and reasons for not taking or having stopped ART from a 7 item multiple choice list and/ or answered an open-ended question to give other reasons. Responses were classified as fear of consequences, perceived lack of need, and ART inaccessibility based on factor analysis. Associations between not taking ART because of fear of consequences, and demographic, behavioural and contextual indicators were identified in a multivariable regression model.

**Results:**

13,353 (7.7%) of 174,209 respondents had been diagnosed with HIV. Among them 3,391 (25.4%) had never received ART, and 278 (2.1%) had stopped taking ART. Perceived lack of need was by far the most common reason for not taking or stopping ART (mentioned by 3259 (88.8%) respondents), followed by fear of consequences (428 (11.7%)), and ART inaccessibility (86 (2.3%)). For all reasons, an East-West gradient could be seen, with larger proportions of men living in Central and Eastern Europe reporting reasons other than medical advice for not taking ART. A minority of men were reluctant to start ART independent of medical advice and this was associated with experiences of discrimination in health care systems.

**Conclusions:**

ART is widely available for MSM diagnosed with HIV across Europe. Not being on treatment is predominantly due to treatment not being recommended by their physician and/or not perceived to be needed by the respondent.

## Introduction

The introduction of combination antiretroviral therapy (ART) in 1996 had a substantial impact on HIV-related morbidity and mortality in all populations with access to treatment [1]. Recommendations on when to start treatment have changed several times and remain controversial. The initial strategy of strong early treatment (“Hit hard and early”) was abandoned around the year 2000 because of serious side effects of the drugs available at that time and increasing evidence that virus eradication would not be possible even with effective long-term treatment. Subsequently, the CD4 cell count was established as a primary parameter to evaluate the need for ART, and international and European treatment guidelines recommended treatment initiation when approaching certain CD4 thresholds. First, a CD4 threshold of 200 to 250 CD4 cells per micro litre was recommended. Around 2008, the threshold was increased to 350, based on evidence of clinical benefits from earlier treatment initiation [2]. Newer drugs also had fewer side effects and improved adherence. Both perceived treatment benefits and fewer side effects contributed to a shift towards earlier treatment initiation. However, controversies about when to start ART have continued, with WHO guidelines recommending treatment at less than 500 CD4 cells, while the U.S. and France have recently removed any CD4 criteria for treatment initiation [3–5]. Other European guidelines remain more conservative, waiting for better randomised controlled trials evidence for earlier treatment initiation [6–8].

Many factors shape treatment guidelines and prescribing practices besides evidence from clinical trials. These include treatment costs, availability of drugs, and support for treatment adherence, availability and qualification of medical personnel, and treatment literacy and demand.

In recent years, international monitoring of national responses to the HIV epidemic (UNGASS and GARP reporting) has revealed substantial differences in ART access across different regions, countries and sub-populations, including in the WHO European region [9]. In this paper we analyse ART coverage, and reasons for never having started or having stopped ART, among HIV-diagnosed men who have sex with men (MSM) in 38 European countries.

## Methods

We used European MSM Internet Survey (EMIS) data. A detailed description of the methods have been published elsewhere [10]. Briefly, EMIS was a community-recruited, anonymous, self-completed online survey conducted simultaneously in 25 languages across 38 countries. No financial incentives were given. No IP addresses were collected. Participants were mainly recruited through online social media for gay and bisexual men. The survey was accessible online from June 6 to August 31, 2010.

### Measures

#### HIV infection and treatment status

All respondents were asked whether they had ever received an HIV test result. All who answered “Yes, I’ve tested positive (I have HIV infection)” were asked the year of their HIV diagnosis, if they had ever started treatment, and if they were still taking it.

#### Reasons for not taking antiretroviral treatment

Those who reported either never having started or having stopped ART were asked why. For both questions (never having started ART, having stopped ART), respondents were asked to indicate as many as apply to them from a list of seven reasons: (1) My doctor says I don't need anti-retroviral treatment at the moment; (2) I feel it is not necessary; (3) To avoid the side-effects; (4) I don't want to be reminded about HIV every day; (5) I'm afraid people will notice; (6) I can’t afford the treatment; (7) The treatment is not available in the country I live in. The reasons were generated from qualitative answers to the same question in an earlier survey and were rotated on presentation to prevent ordering artefacts [11]. An eighth option (“Other reason”) was always presented as the last option which, if checked, prompted the open-ended question, “For what other reason have you never taken (or stopped) antiretroviral treatment?” All qualitative responses were translated to English, content analysed, and either re-coded into existing response options or coded into new response options.

#### Experience with HIV testing and diagnosis

All respondents who had ever tested for HIV were asked how satisfied they were with (a) the way the testing service kept their confidentiality, (b) the respect with which they were treated, and (c) the counselling they received (if any). The answer options were ‘Very satisfied’, ‘Satisfied’, ‘Dissatisfied’, ‘Very dissatisfied’, and ‘I don't remember / I did not think about it’ (plus ‘I did not receive counselling’ for the counselling question). For regression analysis, binary variables (‘Very satisfied’, ‘Satisfied’ = satisfied; ‘Dissatisfied’, ‘Very dissatisfied’ = dissatisfied) were constructed.

#### Experience of HIV-related discrimination

Men with diagnosed HIV were asked to say how often they had been denied medical help because they have HIV. Response options were ‘Never’, ‘Rarely’, ‘Sometimes’, ‘Often’, ‘Very often’, and ‘Does not apply to me’. For regression analysis, “denied medical help” was constructed as a binary variable (never or only rarely denied medical help vs. sometimes or (very) often denied medical help).

#### Regional grouping of countries

For many analyses of the EMIS dataset a grouping in 9 European sub-regions is used [12]. The nine sub-regions are:

- West—Belgium, France, Republic of Ireland, the Netherlands, and the United Kingdom.
- Northwest—Denmark, Finland, Norway, and Sweden.
- Central-West—Austria, Switzerland, Germany, and Luxemburg.
- Southwest—Greece, Spain, Italy, and Portugal.
- Central-East—The Czech Republic, Hungary, Poland, Slovenia, and Slovakia.
- Southeast (EU)—Bulgaria, Cyprus, Malta, and Romania.
- Southeast (non-EU)—Bosnia and Herzegovina, Croatia, Macedonia, Serbia, and Turkey.
- Northeast—Estonia, Lithuania, and Latvia.
- East—Belarus, Moldova, Russia, and Ukraine.

Because the numbers of HIV-diagnosed men and particularly the men not taking ART in eastern European sub-regions were very small, the Central-East, Southeast EU and Southeast non-EU sub-regions were merged (corresponding to the WHO sub-region of Central Europe) as were the East and Northeast sub-regions (corresponding to the WHO sub-region of Eastern Europe), and these two composite sub-regions compared with the four western EMIS sub-regions.

#### Other variables

Education was measured using the six levels of the International Standard Classification of Education (ISCED, 1997 version) corresponding to the educational system of each country. For regression analysis, a binary variable distinguishing between the four lower and the two higher ISCED levels was created.

Outness was defined as the proportion of people known to the respondent (family, friends, and work or study colleagues) who know he is attracted to men, measured on a 5-point scale from “all or almost all” to “none”. For regression analysis, a binary variable was created (“outmost” = all other answers vs. out to most people I know).

Size of settlement was measured on a 5-point scale from “a very big city or town (a million or more people)”, to “a village/the countryside (less than 10,000 people)”. For regression analysis, a binary variable distinguishing between cities with less than 500,000 and 500,000 or more inhabitants was used.

Migrants were defined as men not born in their current country of residence.

### Statistical analysis

#### Grouping of reasons for not taking ART

In order to decide the number of factors to be retained, a principal component analysis (PCA), followed by a parallel analysis was performed. Polychoric correlation was also performed but not presented in the paper as it confirms the results from the factor analysis. The seven specific reasons which were retained were co-correlated to produce a matrix which was analysed with Varimax rotation. Those factors with loadings of 0.4 and above were identified and the items so loaded were then used to interpret the factors.

#### Multivariable regression model

We applied an individual regression model for not taking ART for reasons categorised as “fear of consequences” (as compared to “lack of need”), including variables describing experiences with health care institutions with respect to HIV such as denial of medical help due to HIV status, dissatisfaction with confidentiality, respect, or counselling when receiving an HIV diagnosis, and demographic variables such as sub-region of residence, settlement size, age, education and migration status.

### Ethical approval

The study was approved by the Research Ethics Committee of the University of Portsmouth, UK (REC application number 08/09:21).

## Results

### Descriptive analysis

Out of 174,209 EMIS respondents with valid data, 13,353 (7.7%) reported having diagnosed HIV infection. Of those, 9,484 (71.0%) were currently taking ART, 278 (2.1%) had done so in the past but had stopped taking it, 3,391 (25.4%) had never taken ART, and for 200 (1.5%) treatment status was unclear.

#### Factors associated with ART coverage

Each extra year lived with HIV increased the odds of receiving ART by 21% (OR = 1.21; 95%-CI: 1.19–1.21; Nagelkerke’s Pseudo R^2^ = 22%). No relevant associations were found between receiving ART and recruitment site, education level, employment status, sexual identity, migrancy status, or settlement size.

Lower proportions of diagnosed men were on ART in eastern and central European countries compared to western European countries, probably at least partly related to the later onset of HIV-epidemics among MSM in Central and Eastern Europe (see Table 1).

**Table 1:** ART treatment status by demographic variables and HIV-related health care experiences among MSM living in Europe with diagnosed HIV.

		1 On ART	2 Has stopped taking ART	3 Has never started ART	4 Treatment status unclear	Total
Total		9,484 (100.0%)	278 (100.0%)	3,391 (100.0%)	200 (100.0%)	13353 (100.0%)
Region of residence	1 EMIS West	2,976 (31.4%)	66 (23.7%)	948 (28.0%)	67 (33.5%)	4,057 (30.4%)
	2 EMIS Northwest	334 (3.5%)	10 (3.6%)	86 (2.5%)	8 (4.0%)	438 (3.3%)
	3 EMIS Central-West	3,742 (39.5%)	120 (43.2%)	1,127 (33.2%)	64 (32.0%)	5,053 (37.8%)
	4 EMIS Southwest	1,965 (20.7%)	67 (24.1%)	810 (23.9%)	52 (26.0%)	2,894 (21.7%)
	5 WHO Central Europe	264 (2.8%)	9 (3.2%)	174 (5.1%)	6 (3.0%)	453 (3.4%)
	6 WHO Eastern Europe	203 (2.1%)	6 (2.2%)	246 (7.3%)	3 (1.5%)	458 (3.4%)
Age groups (quartiles)	<25	198 (2.1%)	18 (6.5%)	355 (10.5%)	6 (3.0%)	577 (4.3%)
	25–39	3,793 (40%)	123 (48.2%)	2,099 (61.9%)	62 (31.0%)	6,088 (45.6%)
	40+	5,493 (57.9%)	126 (45.3%)	937 (27.6%)	132 (66.0%)	6,688 (50.1%)
University degree (ISCED 1997)*		4,618 (48.9%)	121 (44.2%)	1,692 (50.2%)	92 (48.2%)	6,523 (49.1%)
Residence in a bigger city (>500,000)*		5,596 (61.1%)	176 (66.4%)	2,108 (63.6%)	109 (58.3%)	7,989 (61.8%)
Out to most people I know		7,637 (81.0%)	230 (83.6%)	2,553 (75.6%)	150 (75.0%)	10,570 (79.6%)
Dissatisfied with confidentiality when testing for HIV*		1,119 (12.6%)	42 (15.9%)	419 (13.1%)	28 (17.7%)	1,608 (12.8%)
Dissatisfied with respect when testing for HIV*		1,287 (14.1%)	50 (18.4%)	439 (13.5%)	31 (18.6%)	1,807 (14.1%)
Dissatisfied with counselling when testing for HIV or no counselling*		3,067 (34.0%)	94 (34.6%)	1,033 (31.7%)	62 (37.1%)	4,256 (33.4%)
Has sometimes or (very) often been denied medical help because of HIV *		891 (9.5%)	40 (14.9%)	191 (5.8%)	20 (11.6%)	1,142 (8.7%)

* due to differences in missing data the absolute number of respondents for each question varies between 12,527 and 13,353.

ISCED = International Standard Classification of Education, 1997 version

#### Reasons for never having started ART

Of the 3,391 respondents who had never taken ART, 3,355 (98.9%) gave at least one reason for not doing so (missing n = 36). Since respondents were allowed to give more than one reason, all reasons sum up to more than 100% and any one respondent may appear under different reasons. The overwhelmingly most common reason, indicated by 87.7%, was that their doctor did not yet recommend it. The next two most common reasons were “I feel it is not necessary” (8.7%) and “To avoid side effects” (6.6%). The least common reason of those offered to respondents was “The treatment is not available in the country I live in” (0.4%; see Table 2).

**Table 2:** ART treatment status and reasons for not taking ART among MSM living in Europe with diagnosed HIV.

	2 Has stopped taking ART	3 Has never started ART	Total
Total	272 (100.0%)	3355 (100.0%)	3627 (100.0%)
Treatment not yet recommended	173 (63.6%)	2,941 (87.7%)	3,114 (85.9%)
I feel it is not necessary	32 (11.8%)	293 (8.7%)	325 (9.0%)
To avoid side effects	78 (28.7%)	221 (6.6%)	299 (8.2%)
I don't want to be reminded about HIV every day	41 (15.1%)	171 (5.1%)	212 (5.8%)
I'm afraid people will notice	14 (5.1%)	84 (2.5%)	98 (2.7%)
Treatment not affordable	9 (3.3%)	71 (2.1%)	80 (2.2%)
Treatment not available	2 (0.7%)	14 (0.4%)	16 (0.4%)
No hope	6 (2.2%)	4 (0.1%)	10 (0.3%)
Unknown ('other', but no explanation)	4 (1.5%)	40 (1.2%)	44 (1.2%)
PEP	5 (1.8%)	0 (0.0%)	5 (0.1%)
Diagnosis too recent / lab results pending	0 (0.0%)	78 (2.3%)	78 (2.2%)
Does not know what antiretroviral treatment is	0 (0.0%)	27 (0.8%)	27 (0.7%)
Distrust in medical science / Duesberg etc.	2 (0.7%)	17 (0.5%)	19 (0.5%)

Answers to the open-ended question, “For what other reason have you never taken antiretroviral treatment?” which could not be re-coded in existing responses revealed three main reasons:

Recent diagnosis/lab results pending: By far the most common “other reason” for never having taken ART was having been diagnosed very recently, not having had time to visit a specialist, being scheduled for treatment but not yet having taken it, or still waiting for some laboratory results to decide whether treatment was indicated. These responses were re-coded as “Recent diagnosis/lab results pending” (n = 78; 2.3%).

Not knowing what ‘anti-retroviral therapy’ is: Some respondents were not aware that there were treatments for HIV infection. It was striking that most answers of this type were given in Dutch, possibly indicating that in the Netherlands and Flanders, a term other than *“anti-retrovirale behandeling”* is used in public health communication.

Distrust in medical science: Finally, some respondents (n = 17; 0.5%) indicated a lack of trust in research or treatment: “*ART—this is more a business than a real need*. *There are other ways to control the viral load but one only speaks about ART*” (Russian response: “АРТ–это скорее бизнес чем реальная необходимость. Есть другие способы контролировать нагрузку но говорят лишь об АРТ.”); or lack of trust in reliable knowledge about HIV infection itself: “*Firmly embrace the dissident theory of HIV*… *And there is evidence that HIV does not cause AIDS*!” (Spanish response: “Abrazo firmemente la teoría disidente del vih… Y pruebas de que vih no es la causa del sida no faltan!”).

No hope: A few men (n = 4; 0.1%) indicated a lack of interest in living (with HIV), or in prolonging their lives: “*I do not want to live with the shame*” (German response: “Ich möchte nicht mit der Schande leben.“) or “*Have lived long enough*” (Hungarian response: Eleget éltem.”). These responses were re-coded as “No hope”.

#### Reasons for having stopped ART

Among men who reported ever having received antiretroviral therapy, 278 said they had stopped taking it. Reasons were given by 272 men (missing n = 6). The most common reason for having stopped—like the most common reason for not having started yet—was a physician’s recommendation (63.6%). However, 28.7% of those who stopped treatment did so because of side effects, and 15.1% indicated fatigue at taking daily pills (“I don’t want to be reminded about HIV every day” (English response)); see Table 2.

After re-coding of open-ended answers into existing response options, other reasons for stopping treatment were provided by 35 respondents. Four of them provided no further explanation. In content analysis, three novel answers emerged:

Research: Some men (n = 18) indicated that they had received treatment as part of a clinical trial they were enrolled in: *“I was on a clinical trial where treatment was given for 48 weeks to those who it could be proved had only recently become HIV-positive*. *That period of treatment has now ended”* (English response). These responses were re-coded as “Research” (n = 18) and subsumed under “treatment not yet recommended”.

PEP: A few men (n = 5) said that the ART they had in the past was post-exposure prophylaxis.

No hope: A few men (n = 6) indicated a lack of interest in living (with HIV), or in prolonging their lives.

Distrust in medical science: Two respondents indicated a lack of trust in research or treatment as reasons for having stopped taking ART.

In total then, twelve reasons and one remaining category of answers who had indicated “other reasons”, but had not given a further explanation were identified for further factor analysis (ART1 " ART not yet needed"; ART2 " To avoid side effects"; ART3 " I feel it is not necessary"; ART4 " I'm afraid people will notice"; ART5 " I don't want to be reminded about HIV every day"; ART6 " ART not available"; ART7 " ART not affordable"; ART8 "Unknown ('other', but no explanation)"; ART9 "PEP (stopped ART)"; ART10 "Lab results not complete (never ART)"; ART11 "Does not know what antiretroviral treatment is (never ART)"; ART12 "Distrust in medical science / Duesberg etc."; ART13 "No hope").

### Grouping of reasons for never taking or having stopped ART

Including the recoded variables ART1-ART13 in the analysis, we obtained a Kaiser-Meyer-Olkin measure of sampling adequacy (KMO) of 0.527 (around the limit to be acceptable). The Bartlett test of Sphericity was acceptable (0.000), and the ALPHA Cronbach was also acceptable (0.697).

Moreover, some items seemed to be strongly inadequate for the factor analysis, for example ART9-ART13 presented the lowest item-rest correlation, which means they have low correlation with the other items and this suggested dropping these items.

In addition, the principal factor analysis indicated ART8 as a weak item (KMO = 0.276 and loading <0.2).

The KMO without ART8-ART13 was 0.707.

Additional theoretical considerations not to include these answers in further factor analysis were:

*ART9 "PEP"*—taking ART for prophylaxis is different from ART for treatment of HIV infection;

*ART10 "Lab results not complete"*—treatment decisions were yet to be made;

*ART11 "Does not know what antiretroviral treatment is (never ART)"*—possible misunderstanding of the question;

For all these reasons we decided to keep only ART1-ART7 and to perform the factor analysis on this set of items. The Kaiser-Meyer-Olkin measure of sampling adequacy for each item is presented in Table 3.

**Table 3:** Kaiser-Meyer-Olkin measures of sampling adequacy for the seven factors retained after principal component analysis.

Treatment not yet recommended	*0*.*717*
To avoid side effects	*0*.*759*
I feel it is not necessary	*0*.*676*
I'm afraid people will notice	*0*.*716*
I don't want to be reminded about HIV every day	*0*.*717*
Treatment not available	*0*.*661*
Treatment not affordable	*0*.*625*

Respondents who had indicated one of the four reasons for which also theoretical considerations argued against inclusion in the factor analysis (i.e. recent diagnosis; not knowing what antiretroviral treatment is; distrust in medical science; PEP—but not ART8 “other reasons” and ART13 “no hope”) were further excluded from multivariable analysis.

The factor analysis performed with varimax rotation suggested to group together the remaining seven reasons into three factors showing a low correlation between them. Loadings for each variable were high and did not cross-load between the factors (with the exception of ‘Side effects’ which loaded on both component 1 and component 2; see Table 4). We labelled the three components as 1) perceived lack of need (includes “my doctor says I don't need ART at the moment”, “I do feel it is not necessary”), mentioned by 3,259 (93.2%) of the eligible respondents; 2) fear of consequences (includes “to avoid the side effects”, “I’m afraid people will notice”, “I don’t want to be reminded about HIV everyday”), mentioned by 428 (12.2%); and 3) ART inaccessible (“I can’t afford the treatment”, “The treatment is not available in the country I live in”), mentioned by 86 (2.5%) of the respondents. The (relatively strong) loading of the reason “To avoid the side effects” on component 1 (lack of need) may reflect the decision to stop treatment because of side effects being taken in conjunction with medical advice. It could also reflect a joint decision of physician and patient to postpone starting ART to avoid side effects.

**Table 4:** Rotated component matrix of reasons for never taking or stopping ART.

	Component 1: lack of need	Component 2: fear of consequences	Component 3: ART inaccessible
Treament not yet recommended	**−0.420**	−0.278	−0.184
To avoid side effects	0.347	**0.385**	0.086
I feel it is not necessary	**0.480**	0.230	−0.043
I'm afraid people will notice	0.134	**0.512**	0.145
I don't want to be reminded about HIV every day	0.276	**0.537**	0.078
Treatment not available	−0.005	0.129	**0.327**
Treatment not affordable	0.070	0.171	**0.405**

Table 5 shows numbers and proportions of men identifying the respective answers, grouped by categories and sub-regions. The rank order of answers was very similar in all sub-regions with minor variations between the western sub-regions. For all reasons retained in further analysis (except “treatment not being recommended”), an East-West gradient was evident (see Table 5). Men living in the WHO Central and Eastern European Regions were more likely than men living in the combined western sub-regions to feel that treatment was not yet necessary, to be afraid of side effects, to be unwilling to be reminded about HIV every day, or to be afraid of people noticing they have HIV. They were also more likely to state that they could not afford treatment, or that the treatment was not available in the country where they lived. Fear of side effects was very similar in the EMIS Central-West and WHO Central European region, and the reason “I don’t want to be reminded about HIV everyday” was as frequent in EMIS Northwest and EMIS Central-West as in the WHO Central European region.

**Table 5:** Regional distribution of HIV-related health care experiences and reasons for not taking ART.

		Region of residence						Total
		EMIS West	EMIS Northwest	EMIS Central-West	EMIS Southwest	WHO Central Europe	WHO Eastern Europe	
		N (%)	N (%)	N (%)	N (%)	N (%)	N (%)	N (%)
	**Full N**	**4,006**	**433**	**5,023**	**2,863**	**448**	**451**	**13,224**
Being out	Out to most people I know	3.439 (86.1%)	366 (85.3%)	4,230 (84.7%)	1,974 (69.5%)	249 (56%)	216 (48.2%)	10,474 (79.7%)
Satisfaction with confidentiality when testing for HIV	Dissatisfied	404 (10.8%)	49 (12.3%)	516 (10.9%)	426 (15.6%)	90 (22.2%)	101 (24.8%)	1,586 (12.8%)
Satisfaction with respect when testing for HIV	Dissatisfied	536 (13.9%)	66 (15.9%)	510 (10.7%)	494 (17.8%)	97 (22.7%)	85 (20.3%)	1,788 (14.1%)
Satisfaction with counselling when testing for HIV	Dissatisfied or no counselling	1,679 (44.4%)	104 (25.4%)	1,176 (24.6%)	947 (34.1%)	139 (32.6%)	163 (38.7%)	4,208 (33.4%)
Denied medical help	Sometimes or often medical help denied because of HIV	304 (7.7%)	27 (6.3%)	460 (9.3%)	181 (6.5%)	84 (19.03%)	82 (18.7%)	1,138 (8.8%)
								
**Reasons for not taking ART**	**N**	**955**	**89**	**1197**	**842**	**172**	**243**	**3498**
	Treatment not yet recommended	858 (89.8%)	81 (91.0%)	1,055 (88.1%)	785 (93.2%)	149 (86.6%)	178 (73.3%)	3,106 (88.8%)
	I feel it is not necessary	85 (8.9%)	2 (2.2%)	114 (9.5%)	36 (4.3%)	17 (9.9%)	60 (24.7%)	314 (9.0%)
	To avoid side effects	75 (7.9%)	5 (5.6%)	119 (9.9%)	42 (5.0%)	16 (9.3%)	35 (14.4%)	292 (8.3%)
	I don't want to be reminded about HIV every day	55 (5.8%)	6 (6.7%)	77 (6.4%)	28 (3.3%)	11 (6.4%)	34 (14.0%)	211 (6.0%)
	I'm afraid people will notice	23 (2.4%)	1 (1.1%)	31 (2.6%)	19 (2.3%)	8 (4.7%)	16 (6.6%)	98 (2.8%)
	Treatment not affordable	12 (1.3%)	1 (1.1%)	36 (3.0%)	7 (0.8%)	8 (4.7%)	13 (5.3%)	77 (2.2%)
	Treatment not available	5 (0.5%)	0 (0.0%)	1 (0.1%)	1 (0.1%)	1 (0.6%)	7 (2.9%)	15 (0.4%)

### Multivariable regression model

#### Reasons for not taking or having stopped ART—Fear of consequences and experiences of bad healthcare

In univariable analysis, reasons for not taking ART or having stopped taking ART with high factor loadings on component 2 were significantly more frequent among men who reported having been denied medical help due to their HIV status, and among men who reported having been dissatisfied with confidentiality, respect, or counselling when receiving their HIV diagnosis. In multivariable analysis, controlling for age, education, settlement size, migration status, country/region of residence, and outness, factors significantly correlated with ‘fear of consequences’ reasons for not taking ART were European sub-region, having been denied medical help, and dissatisfaction with confidentiality and counselling when receiving the positive HIV test result (see Table 6).

**Table 6:** Uni- and multivariable analysis of factors associated with “fear of consequences” (component2) as reason for not taking ART.

	Univariable	Multivariable	
	OR (95%CI)	OR (95%CI)	Significance
Dissatisfaction with confidentiality	**2.21 (1.70–2.87)**	**1.49 (1.05–2.12)**	0.028
Dissatisfaction with respect	**1.95 (1.50–2.50)**	1.13 (0.78–1.62)	0.530
Dissatisfaction with counselling	**1.96 (1.59–2.42)**	**1.67 (1.28–2.17)**	<0.001
Denied medical help	**2.82 (2.05–3.87)**	**2.24 (1.57–3.21)**	<0.001
EMIS region Central-West	reference	reference	.000
EMIS region West	**0.72 (0.55–0.94)**	**0.71 (0.53–0.94)**	0.018
EMIS region Northwest	0.67 (0.33–1.36)	0.61 (0.26–1.44)	0.257
EMIS region Southwest	**0.50 (0.37–0.67)**	**0.47 (0.34–0.65)**	<0.001
WHO region Central Europe	0.92 (0.58–1.47)	0.88 (0.53–1.45)	0.608
WHO region East Europe	**1.79 (1.27–2.51)**	1.43 (0.96–2.12)	0.079
Constant		**0.02**	<0.001

## Discussion

Among MSM living in Europe and diagnosed with HIV, the overwhelmingly most common reason for not taking ART was a lack of need, based on advice of care providers. Lack of affordability or availability of ART was rare. Reasons for not taking ART that can be termed ‘fear of the consequences’ were associated with having previously experienced inadequate health care.

Since the EMIS data was collected in 2010, there has been some return to the “hit hard, hit early” approach. The European HIV Treatment Guidelines have recently been updated to recommend that the use of ART should be considered and actively discussed with HIV-positive patients irrespective of their CD4 level [13]. This recommendation is intended to reflect the undefined risk/benefit ratio for use of ART with CD4 cell counts above 350/μl. The CD4 cell count unrestricted U.S. and French recommendations are based both on the assumption that HIV does substantial damage to the immune system in primary infection, and on an increasing acknowledgment that starting treatment substantially reduces infectivity. Data on the uptake of treatment by MSM diagnosed with HIV and CD4 cell counts above 350 or 500 cells/μl and physicians’ attitudes and practices regarding early treatment initiation across Europe after these guideline changes have not yet been published. Continued monitoring will be needed to assess the effects of the changing guidelines on treatment uptake.

Experiences reported from San Francisco after adopting a policy of offering ART to all HIV-infected persons regardless of CD4 cell count demonstrate increasing ART uptake among persons with higher CD4 cell count. However, racial and social disparities regarding ART uptake persisted [14].

A recently presented survey among HIV-infected patients from the European Union and Australia revealed reasons for delaying the start of ART very similar to our findings. Among the 508 HIV-positive patients surveyed, 92% had a CD4 cell count higher than 350 cells/μl (the current threshold at which ART is recommended). The most common reasons for not starting /delaying ART were “I rely on my body to tell me when to start” and “I’m waiting until symptoms occur” and resemble our “I feel it is not necessary” reason. Not wanting to be reminded about their HIV status was indicated by 47% of the respondents in this study. Notably, considerable proportions of physicians, who were also surveyed in the same study, were reluctant to recommend treatment to their patients in certain circumstances, even if the CD4 cell count was already below 350 cells/μl. One of the reasons was if the patient was too depressed [15].

In our study, lack of availability or affordability of ART was reported infrequently (<3%) as a reason for not taking ART. While treatment rates for HIV-infected intravenous drug users are stubbornly low in Eastern Europe [16], to a large part probably due to non-availability of oral opioid substitution treatment, there seem to be no structural barriers to treatment for men who acquired HIV homosexually.

Approximately 15% of the HIV-positive respondents who are currently not taking ART mention reasons we have grouped as “fear of consequences”. Reasons for non-treatment other than medical advice are reported more often by men living in non-EU countries of east and Southeast Europe. Greater concerns regarding side effects may reflect higher rates of side effects due to more frequent use of less convenient treatment regimens and less experienced HIV care providers, a worse image of ART in mass media communication in these countries, and lower treatment literacy due to less accessible treatment information. Other reasons for non-treatment are related to HIV-stigma (fear of being recognised as HIV infected), internalised HIV-stigma (not wanting to be reminded of HIV), and internalised homonegativity [17, 18]. The association of non-treatment with inadequate health care (denial of medical help due to HIV status, poor experiences of HIV diagnosis) points to the adverse effects of HIV- and gay-related stigma on treatment preparedness and adherence among HIV-infected MSM.

### Limitations

EMIS collected no data on current CD4 count or CD4 count at treatment initiation. Thus, we cannot exclude the possibility that differences in treatment rates between countries or European sub-regions are related to variations in the immunological status of HIV infected individuals. Migrant MSM without legal status in the European Union would be one group which may have difficulties in accessing health care in many European countries. This group may be underrepresented in the study sample, which consists of men with online access. Although it seems plausible that the correlation between inadequate experiences of health care and not wanting to take treatment because of a fear of the consequences is causal, both could be independent consequences of another, unmeasured cause. It should be noted that the survey questions covered only a few selected aspects of individual interactions with the health care system.

## Conclusions

Antiretroviral therapy is widely available for MSM diagnosed with HIV across Europe. Assuming that medical advice is mostly based on treatment guidelines, absence of treatment taking appears predominantly due to not meeting the treatment guideline for starting ART. The consequences of recent guideline changes on treatment uptake should be monitored. A minority of men diagnosed with HIV appear to be reluctant to start ART in spite of medical advice to do so. This appears to be related to experienced or expected HIV- or gay-related discrimination in society and the health care systems, which may undermine the trust of people with HIV in medical advice. An additional and related factor is the fear that taking ART makes it harder to avoid disclosure of HIV infection to friends, relatives and co-workers.

## Supporting Information

S1 Datafile This is the EMIS datafile used for analysis as a csv-file. (CSV)

S1 Codebook This is a txt-file containing descriptions of the variables and analysis syntaxes (SPSS and STATA). (TXT)
